# Research trends and hotspots of exercise therapy in Panvascular disease: A bibliometric analysis

**DOI:** 10.1097/MD.0000000000035879

**Published:** 2023-11-10

**Authors:** Xi Xu, Xiao-Dan Xu, Yin Liang, Tao Xu, Fu-Rong Shao, Lin Zhu, Kun Ren

**Affiliations:** a College of Nursing, Anhui University of Chinese Medicine, Hefei, Anhui, PR China; b Department of Pathology, The First Affiliated Hospital of Anhui Medical University, Hefei, Anhui, PR China; c The First School of Clinical Medicine, Guangdong Medical University, Zhanjiang, Guangdong, PR China; d Anhui Province Key Laboratory of Research & Development of Chinese Medicine, Hefei, PR China; e Institute of Clinical Medicine, The Second Affiliated Hospital of Hainan Medical University, Haikou, Hainan, PR China.

**Keywords:** bibliometrics, CiteSpace, exercise therapy, Panvascular disease, research hotspots, visualized analysis, VOSviewer

## Abstract

Panvascular diseases are a group of vascular system diseases, mainly including the heart, brain, neck, and other parts of the vascular lesions. As a non-pharmacological intervention, exercise therapy could prevent and treat Panvascular diseases. However, few bibliometric analyses of exercise therapy in Panvascular disease exist. This study aimed to analyze the trends and hotspots over the past decade and provide insights into the latest state of the art in global research, thereby contributing to further research in the field. We systematically searched the Web of Science Core Collection (WOSCC) for articles on exercise therapy and Panvascular disease. The acquired information from the reports was analyzed using CiteSpace and VOSviewer software to assess and forecast this field hottest areas and trends. The final analysis included 294 articles by our specified inclusion criteria. The number of publications has gradually increased over the past decade. Stroke was one of the most studied Panvascular diseases. China and the University of Sao Paulo were the country es and institutions that contributed the most to the field. Mary M. McDermott was the most prolific researcher, and the *Journal of Vascular Surgery* published the most articles. The 6-minute walk test, skeletal muscle, oxidative stress, and supervised exercise therapy were hot topics from 2019 to 2023. In conclusion, exploring exercise therapy programs and exercise mechanisms for Panvascular diseases has been ongoing. This study revealed the current status and trends of research in the field and identified hot topics. It was helpful for scholars to understand exercise therapy critical role in treating and preventing Panvascular diseases and provided a reference for clinical decision-making and further research.

## 1. Introduction

Panvascular diseases were a group of systemic vascular diseases with atherosclerosis as a common pathological feature, mainly affecting vital organs such as the heart, brain, kidney, extremities, and large arteries. This concept involved all the vascular systems of the body, including the complex network of arteries, veins, and lymphatic vessels.^[1]^ In 2002, Peter Lanzer and Eric J. Topol first proposed the concept of “Panvascular disease based on the unified understanding of vascular disease as a group of systemic diseases, thus laying the foundation for the discipline of “Panvascular medicine.”^[2]^ Panvascular medicine was an emerging discipline that focused on arteriosclerotic cardiovascular disease (ASCVD), represented by ischemic heart disease, ischemic stroke, and peripheral arterial disease (PAD).^[1]^ ASCVD-related conditions remained the leading cause of morbidity and mortality globally,^[3]^ and the prevalence and associated economic burden were expected to continue to rise.^[4,5]^ Compared with traditional medicine, Panvascular medicine focuses more on the unity of structure and function. It explored the relationship between the development of ASCVD and the macroscopic environment of the body. Atherosclerotic lesions were not usually confined to a single vascular bed; patients with Panvascular disease often had a more extensive atherosclerotic burden.^[6,7]^ The study showed that, in patients with carotid artery disease or PAD, the prevalence of various types of multi-vascular diseases increased, and the prognosis worsened.^[8]^

In recent years, the development and application of exercise therapy in clinical and rehabilitation have been remarkable. Exercise therapy has a wide range of clinical applications, including managing chronic pain and improving the prognosis of patients with cancer and neurological diseases.^[9–11]^ Physical activity independently reduced the risk of Panvascular illness and lowered other cardiovascular risk factors.^[12,13]^ Exercise therapy was found to reduce carotid artery middle thickness in patients with coronary artery disease (CAD),^[14]^ improve proprioception and spasticity in stroke patients^[15]^; reduce arterial stiffness in PAD patients, and improve exercise tolerance, cardiorespiratory capacity, and muscle strength.^[16]^ In addition, exercise interventions modulated cellular oxidative stress and inflammation,^[17,18]^ reduced cell adhesion,^[19]^ and prevented foam cell formation,^[20]^ all of which played a critical role in the development of Panvascular disease. Furthermore, swimming could activate autophagy in AS mice, improve the structural disorder of the artery, and reduce plaque area.^[21,22]^ Exercise training also could reduce superoxide, lipid peroxidation, and inflammatory factor production, ultimately reducing the infarct zone in mice with myocardial infarction (MI).^[23,24]^

Although exercise therapy could effectively improve the quality of life for patients with Panvascular disease in their management and treatment, its protective role in Panvascular disease has also been praised by many guidelines and expert consensus. However, the detailed mechanisms involved in exercise therapy and different exercise prescriptions for patients still require further research. With the increasing number of studies on exercise interventions for Panvascular disease, there was an urgent need for a comprehensive and visual analysis of this field to identify research trends and hot spots. Therefore, we conducted a bibliometric study of exercise therapy for Panvascular disease and mapped out the research landscape, thus revealing the forefront developments in this field.

## 2. Methods

### 2.1. Data sources and search strategy

**Figure 1.**
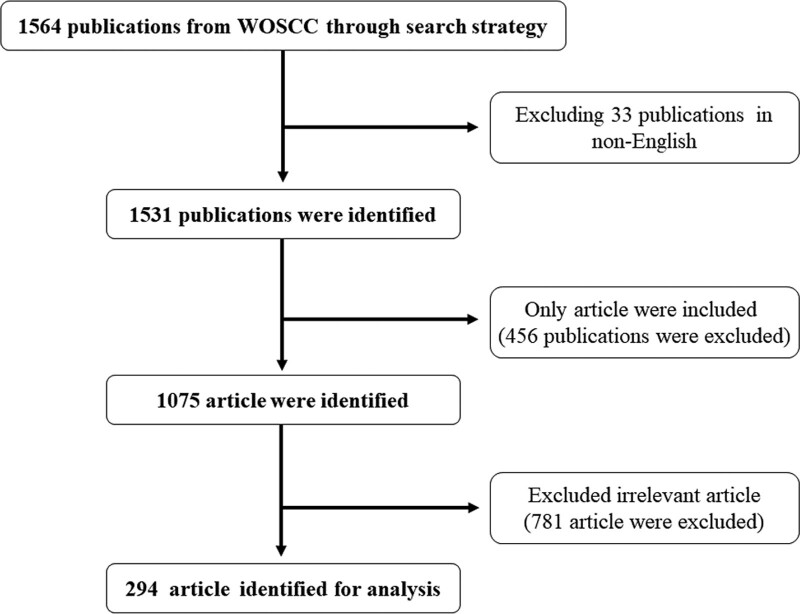
Flow chart of article selection included in this study.

The data for the bibliometric analysis used in this study were collected from the Web of Science Core Collection (WOSCC) database, as WOSCC was considered the most influential database. The period of this study was: June 2013-June 2023. The search strategy was as follows: (“atherosclerosis” OR “Peripheral artery disease” OR “Coronary Artery Disease” OR “myocardial infarction” OR “lower extremity arterial disease” OR “Cerebral Infarction” OR “cerebral ischemic stroke” OR “transient ischemic attack” OR “carotid disease” OR “arterial calcification” OR “arteriosclerosis” OR “stroke”) AND (“exercise therapy” OR “physical exercise” OR “exercise prescription” OR “Exercise rehabilitation” OR “Athletic rehabilitation” OR “Exercise intervention therapy” OR “Motorpathy” OR “Sports treatment”). The main types of literature chosen for this study were articles. Choose “English” as the language type; publications in languages other than English were excluded. Selected papers were downloaded on May 30, 2023. Two researchers independently read the titles and abstracts to exclude irrelevant literature, and 294 articles were finally retained. The screening process is shown in Figure 1.

### 2.2. Bibliometric analysis and visualization

This study used VOSviewer (1.6.19) and CiteSpace (6.2.2) software for bibliometrics and visual analysis. CiteSpace was a JAVA-based citation visualization software widely used in bibliometric studies.^[25,26]^ The analysis of co-cited authors, co-cited journals, timeline views, journal, and citation bipartite overlays, and keyword citation burst analysis in this study was implemented by CiteSpace software. Another bibliometric software was VOSviewer, which converted large amounts of bibliographic data into a visual representation.^[27]^ We used VOSviewer to visualize the network, including co-occurrence analysis of countries, keywords, journals, and institutions. The included literature was exported from WOSCC in plain text format, including complete records and references, and then imported into VOSviewer and CiteSpace for bibliometric analysis.

## 3. Results

### 3.1. Bibliometric analysis of publication in the timeline

**Figure 2.**
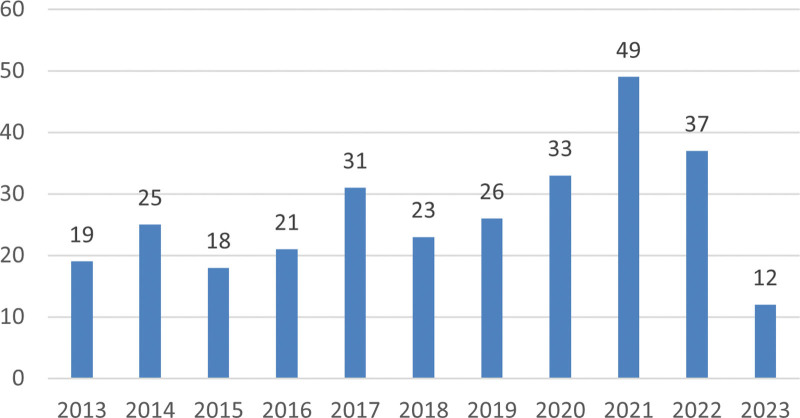
The number of publications per yr from 2013 to 2023.

From June 2013 to June 2023, 294 publications met our inclusion criteria. Studies on exercise therapy in Panvascular disease steadily increased, indicating considerable interest in this research topic. Despite some minor fluctuations, there was an overall upward trend in the number of studies (Fig. 2). In particular, the number of published articles reached 49 in 2021, a decade high.

### 3.2. Bibliometric analysis of countries and institutions

**Figure 3.**
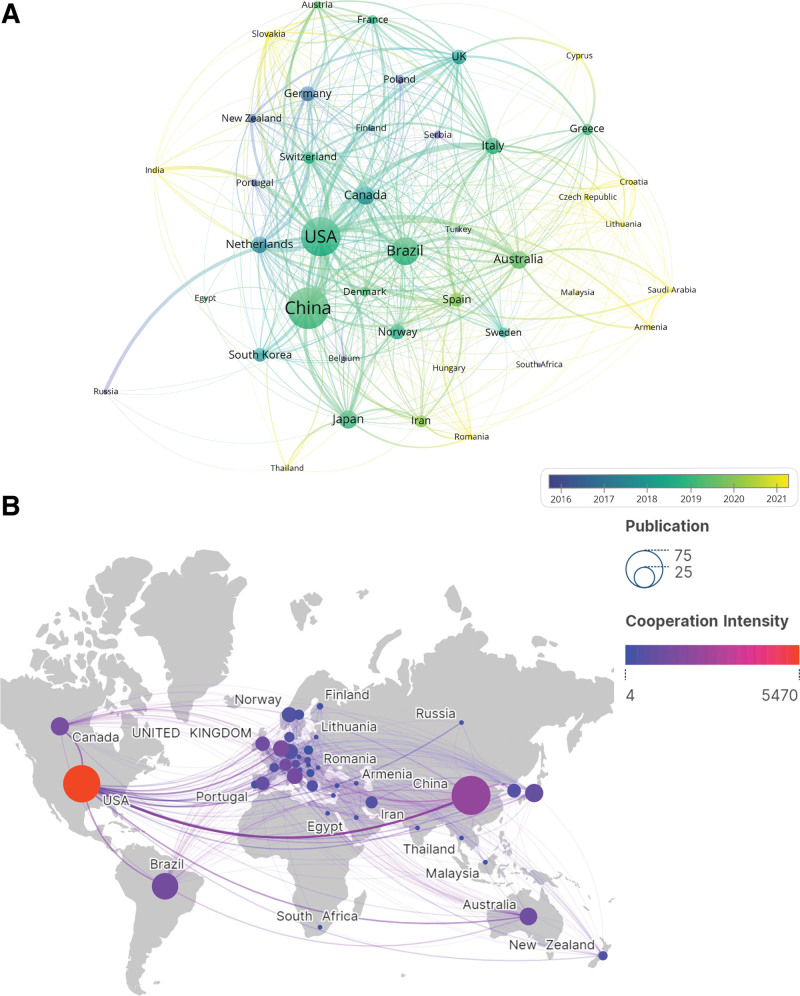
Contribution of different countries to research in exercise therapy for Panvascular disease. (A) The time overlay map of countries cooperation networks. (B) The world map of the intensity of cooperation between countries.

The VOSviewer analysis showed that the literature came from 42 countries and 642 institutions. China (74, 25.17%) had the most published articles (Fig. 3A and Table 1), followed by the United States (68, 23.12%), Brazil (34, 11.56%), Canada (16, 5.44%) and Japan (16, 5.44%). The high centrality of the United States (0.86) represented its crucial position in this field. Despite the Netherlands ranking seventh in total papers, but achieved the first average citations per item, indicating the exceptional quality of Dutch research in the field of exercise therapy in Panvascular disease and its widespread recognition among academics. Figure 3A shows a time overlay map of countries’ cooperation networks. The size of the nodes indicated the number of publications from various countries, the node color represented the change of the corresponding time, and the lines connecting the nodes depicted the connections between countries. New Zealand, Germany, Poland, Serbia, and Portugal were among the first countries to initiate research on the application of exercise therapy in Panvascular diseases, and a large number of articles were published in the early stages. In terms of cooperation intensity (Fig. 3B), the USA had the highest Total Link Strength (5470), indicating that the USA had the highest intensity of cooperation with other countries, followed by China (2209), the Netherlands (1436), Canada (1425) and Switzerland (1260).

**Table 1:** The top 10 countries with the most research results in exercise therapy for Panvascular disease.

Rank	Country	Publication (%)	ACI	TLS	Centrality
1	China	74 (25.17)	15.24	2209	0.1
2	USA	68 (23.12)	18.97	5470	0.86
3	Brazil	34 (11.56)	7.35	1224	0
4	Canada	16 (5.44)	17.69	1425	0.01
=4	Japan	16 (5.44)	18.38	901	0.2
6	Australia	15 (5.10)	12.47	1196	0.04
7	Netherlands	13 (4.42)	19.31	1436	0.04
8	Italy	12 (4.08)	15.83	1248	0.02
9	Germany	11 (3.74)	16.45	523	0.42
=9	Norway	11 (3.74)	13.91	447	0.15

ACI = average citations per item, TLS = total link strength.

**Figure 4.**
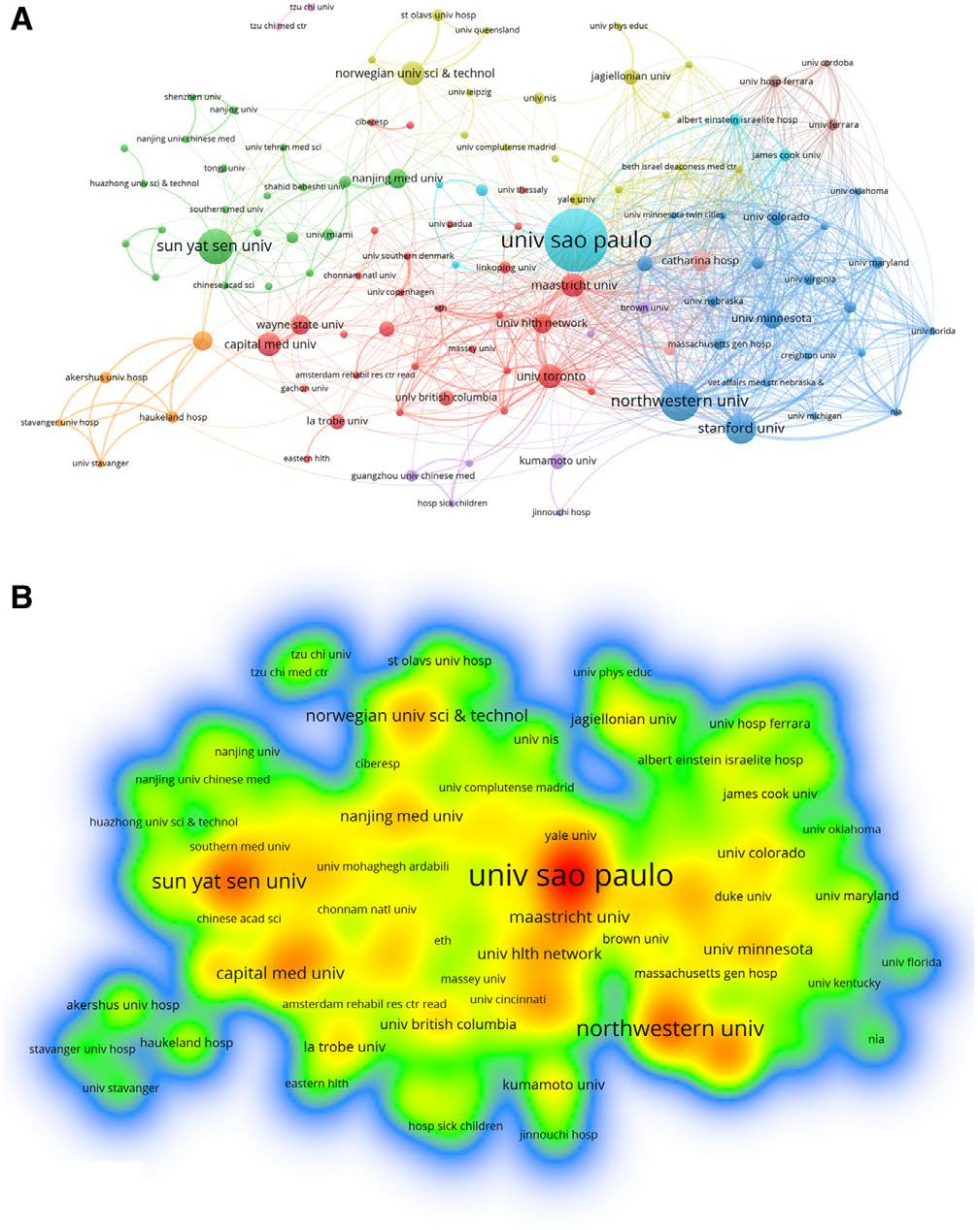
Contribution of different institutions to research in exercise therapy for Panvascular disease. (A) Network diagram, (B) Densitymap of institutions.

Meanwhile, we mapped the network of institutional cooperation in this field (Fig. 4 and Table 2). Institutions with close cooperation could be divided into 8 categories, in which different colors represented clusters of close relationships. Table 2 lists the ten institutions with the highest number of publications. The top 10 institutions were from China (3/10), the USA (2/10), the Netherlands (2/10), Brazil (1/10), Canada(1/10) and Norway(1/10). São Paulo University(16, 5.44%) is the most productive institution, followed by Northwestern University (10, 3.40%), Sun Yat-Sen University (9, 3.06%), Stanford University (8, 2.72%), and Capital Medical University (6, 2.04%).

**Table 2:** The top 10 institutions with the most research results in this field.

Rank	Institutions	Country	Publication (%)	ACI	TLS	Citations
1	University of São Paulo	Brazil	16 (5.44)	4.81	1051	77
2	Northwestern University	USA	10 (3.40)	13.90	3356	139
3	Sun Yat-Sen University	China	9 (3.06)	17.67	213	159
4	Stanford University	USA	8 (2.72)	4.88	2240	39
5	Capital Medical University	China	6 (2.04)	18.00	448	108
6	Maastricht University	Netherlands	6 (2.04)	20.33	1449	122
7	Norwegian University of Science and Technology	Norway	6 (2.04)	15.50	472	93
8	University of Toronto	Canada	6 (2.04)	9.83	1295	59
9	Catharina Hospital	Netherlands	5 (1.70)	8.60	1138	43
10	Nanjing Medical University	China	5 (1.70)	16.00	390	80

ACI = average citations per item, TLS = total link strength.

### 3.3. Bibliometric analysis of authors and co-cited authors

**Figure 5.**
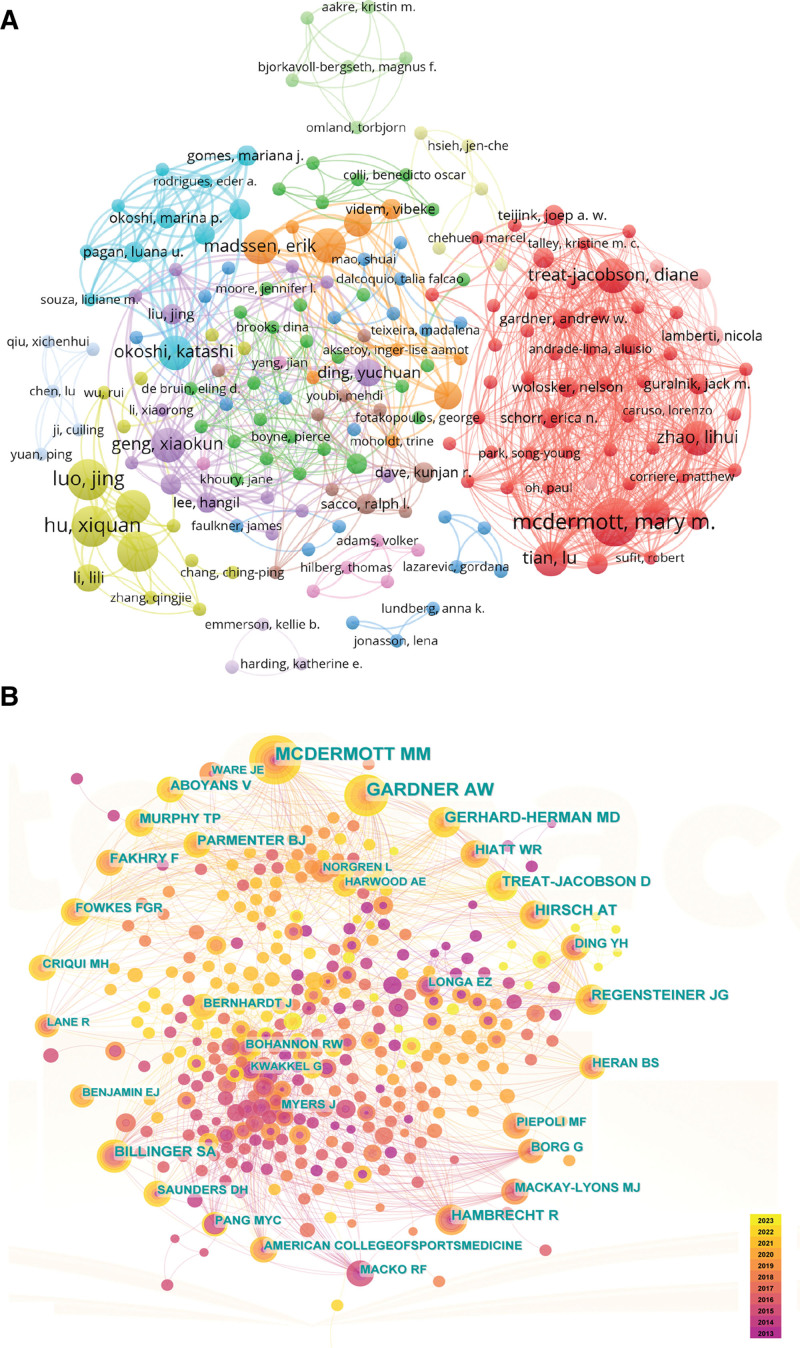
The network of authors and co-cited authors.

According to the author analysis, 1858 authors participated in this field, and 17 authors published more than 4 papers (Fig. 5A and Table 3). Mary M McDermott published the most significant number of studies (7, 2.38%), followed by Xiquan Hu (6, 2.04%), Jing Luo (6, 2.04%), Liying Zhang (6, 2.04%), and Xiaokun Geng (5, 1.70%). Mary M. McDermott from Northwestern University emerged as the most prolific author in research on exercise therapy for Panvascular disease. Her groundbreaking contributions garnered significant attention, evident through her highest total link strength and highly cited. The average number of citations per paper Jing Luo was in first place, which had a certain academic impact.

**Table 3:** The top 10 authors and co-cited authors.

Rank	Author	Publication (%)	ACI	TLS	Co-cited author	Co-citation	Centrality
1	Mary M McDermott	7 (2.38)	10.2857	10241	Gardner Andrew W	38	0.07
2	Xiquan Hu	6 (2.04)	20	2284	Mary M McDermott	34	0.09
3	Jing Luo	6 (2.04)	26	1946	Alan T Hirsch	18	0.07
4	Liying Zhang	6 (2.04)	20	2284	Gerhard-Herman MD	17	0.02
5	Xiaokun Geng	5 (1.70)	15.6	3408	Sandra A Billinger	16	0.12
6	Erik Madssen	5 (1.70)	11.4	1704	Rainer Hambrecht	15	0.12
7	Katashi Okoshi	5 (1.70)	10	5183	Judith G. Regensteiner	15	0.03
8	Lu Tian	5 (1.70)	5.4	6231	Diane Treat-Jacobson	15	0.02
9	Diane Treat-Jacobson	5 (1.70)	9.8	5930	William R Hiatt	13	0.01
10	Rune Wiseth	5 (1.70)	11.4	1704	Victor Aboyans	13	0.01

ACI = average citations per item, TLS = total link strength.

Co-cited authors referred to authors cited simultaneously in different publications articles. Among the 406 authors, 24 were co-cited more than ten times, and only 2 were co-cited over 30 times (Fig. 5B and Table 3). The most co-cited authors were Gardner Andrew W (38), followed by Mary M. McDermott (34), Alan T. Hirsch (18), Gerhard-Herman MD (17), and Sandra A. Billinger (16). Sandra A. Billinger and Rainer Hambrecht tied for first place in terms of centrality, meaning they played an essential bridging role in the research of exercise therapy in Panvascular diseases. Gardner Andrew W. from the University of Oklahoma Health Sciences Center stood out with the highest co-citation frequency, surpassing expectations given his relatively low number of publications. This remarkable achievement attested to the widespread recognition and citation of his high-quality research by scholars and highlighted his high academic reputation. We also observed that 4 out of 10 prolific authors were from China; however, none have received high citations. This could be attributed to the scarcity of representative core research groups or figures and the absence of high-quality, comprehensive studies on exercise therapy for Panvascular disease in China.

### 3.4. Bibliometric analysis of co-cited references

References were crucial in research as they provided the basis for scholarly investigations. By conducting reference co-citation analysis, we could understand the evolution of the body of knowledge in the field and identify current research focus areas. Our study of the co-cited references found that 10,331 references were cited in 294 publications. A total of 20 references were co-cited more than ten times, and Table 4 shows the top ten co-cited references. The most co-cited reference was “Optimal Exercise Programs for Patients with Peripheral Artery Disease: A Scientific Statement from the American Heart Association,” written by Diane Treat-Jacobson et al in January 2019 and published in *Circulation*.^[28]^ This scientific statement provided an overview of the evidence on a wide range of exercises for patients with PAD, which also provided an overall guide for understanding exercise therapy and PAD, as well as research on the optimal exercise regimen for PAD patients.^[28]^ The second co-cited reference was published in the *JAMA-Journal of the American Medical Association* written by Mary M. McDermott et al in January 2009.^[29]^ This randomized controlled clinical trial demonstrated that a 6-month supervised treadmill exercise intervention improved 6-minute walk and treadmill walk performance, increased brachial artery flow-mediated dilatation, and improved quality of life in patients with PAD with or without claudication, and 6 months of resistance training increased maximal treadmill walk distance and stair climbing ability.^[29]^ The third co-cited paper was “Exercise-based cardiac rehabilitation for coronary heart disease” by Heran et al in July 2011.^[30]^ This systematic review examined the effects of exercise-based cardiac rehabilitation on various outcomes. The findings suggested that exercise-based cardiac rehabilitation could reduce overall mortality, cardiovascular mortality, and readmission rates.^[30]^

**Table 4:** The top 10 co-cited references.

Rank	Co-cited reference	Frequency	IF(2022)	Centrality
1	treat-jacobson d, 2019, circulation, v139, pe10, doi 10.1161/cir.0000000000000623	18	37.80	0.05
2	mcdermott mm, 2009, jama-j am med assoc, v301, p165, doi 10.1001/jama.2008.962	18	120.71	0.38
3	heran bs, 2011, cochrane db syst rev, doi 10.1002/14651858.cd001800	17	8.40	0.06
4	gardner aw, 1991, med sci sport exer, v23, p402	17	4.10	0.01
5	fowkes fgr, 2013, lancet, v382, p1329, doi 10.1016/s0140-6736(13)61249-0	15	168.90	0.03
6	billinger sa, 2014, stroke, v45, p2532, doi 10.1161/str.0000000000000022	14	8.30	0.06
7	longa ez, 1989, stroke, v20, p84, doi 10.1161/01.str.20.1.84	14	8.30	0.01
8	mcdermott mm, 2013, jama-j am med assoc, v310, p57, doi 10.1001/jama.2013.7231	13	120.71	0.03
9	gardner aw, 2011, circulation, v123, p491, doi 10.1161/circulationaha.110.963066	13	37.80	0.04
10	fakhry f, 2012, j vasc surg, v56, p1132, doi 10.1016/j.jvs.2012.04.046	12	4.30	0.13

IF = impact factor, JCR = journal citation reports.

### 3.5. Bibliometric analysis of journals and co-cited journals

**Figure 6.**
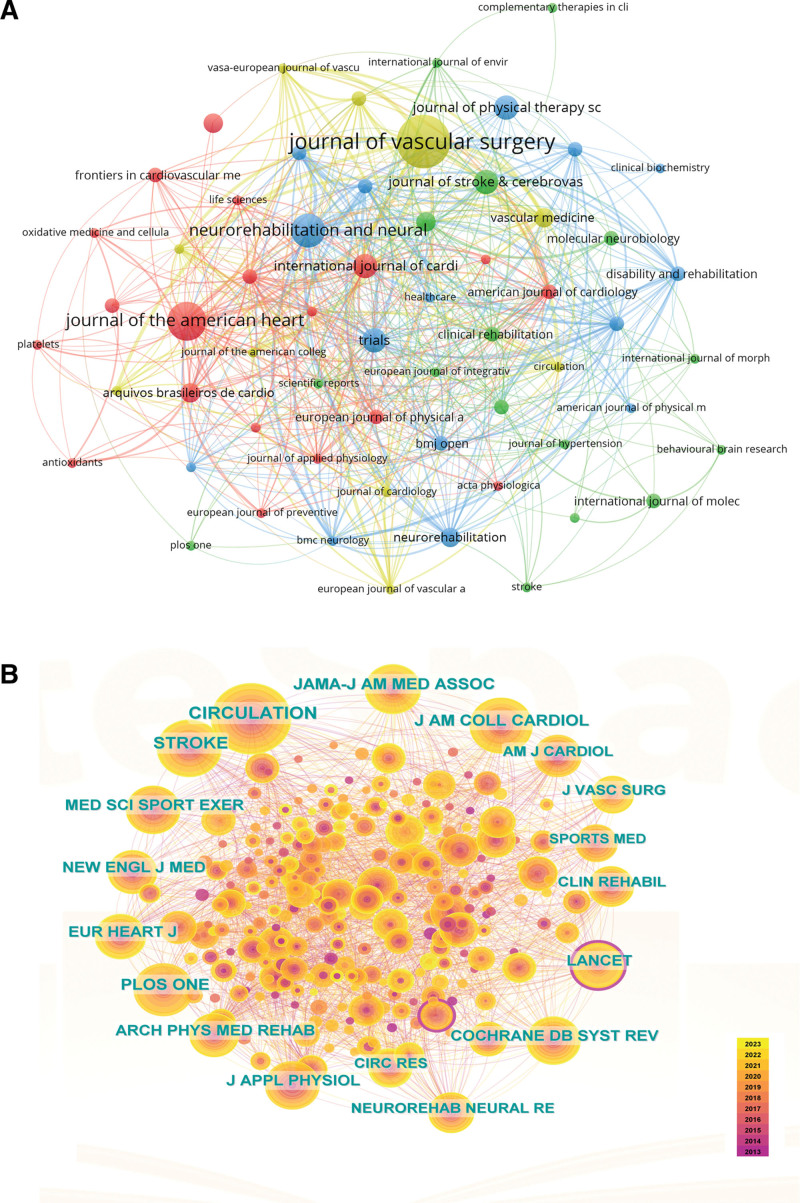
The network of journals and co-cited journals.

176 academic journals published references on exercise therapy in Panvascular disease (Fig. 6A and Table 5). The top 5 journals by the number of publications were the *Journal of Vascular Surgery* (11, 3.74%), *Journal of the American Heart Association* (8, 2.72%), *Neurorehabilitation and Neural Repair* (7, 2.38%), *International Journal of Cardiology* (5, 1.70%), and *Journal of Stroke & cerebrovascular diseases* (5, 1.70%). The average impact factor (IF) of the 10 most productive journals was 3.58. 2 of these journals belonged to the Q1 division, and 4 to the Q2 division. Meanwhile, VOSviewer found 2388 co-cited journals in the past decade (Fig. 6B and Table 4), and 17 journals had citations over 100. The analysis of co-cited journals showed that the most co-cited Journal was *Circulation, followed by Stroke, Journal of the American College of Cardiology, Journal of Vascular Surgery*, and *Archives of Physical Medicine and Rehabilitation* (Fig. 6B and Table 5). Among the top 10 cited journals, the *JAMA-Journal of the American Medical Association* (IF = 120.71) had the highest IF, denoting its recognition as one of the most prestigious and influential medical journals. It has a long-standing history of publishing high-quality research articles, clinical studies, and reviews across various medical disciplines. The average IF of the top 10 cited journals was 21.47. Four co-cited journals having an IF value >5, 7 were in the Q1 district of journal citation reports, indicating that high-quality journals were frequently cited.

**Table 5:** The top 10 journal and co-cited journals.

Rank	Journal	IF(2022)	JCR	Co-cited Journal	IF(2022)	JCR
1	Journal of vascular surgery	4.30	Q2	Circulation	37.80	Q1
2	Journal of the American Heart Association	5.40	Q1	Stroke	8.30	Q1
3	Neurorehabilitation and Neural Repair	4.20	Q1	Journal of the American College of Cardiology	24.00	Q1
4	International Journal of Cardiology	3.50	Q2	Journal of vascular surgery	4.30	Q2
5	journal of Stroke & cerebrovascular diseases	2.50	Q4	Archives of Physical Medicine and Rehabilitation	4.30	Q1
6	Trials	2.50	Q2	JAMA-Journal of the American Medical Association	120.71	Q1
7	Arquivos Brasileiros de Cardiologia	2.60	Q4	Medicine & Science in Sports & Exercise	4.10	Q1
8	Frontiers in Neurology	3.40	Q2	PLoS One	3.70	Q2
9	Journal of the American Heart Association	5.40	Q4	Journal of Applied Physiology	3.30	Q2
10	Neurorehabilitation	2.00	Q4	Neurorehabilitation and Neural Repair	4.20	Q1

IF = impact factor, JCR = journal citation reports.

**Figure 7.**
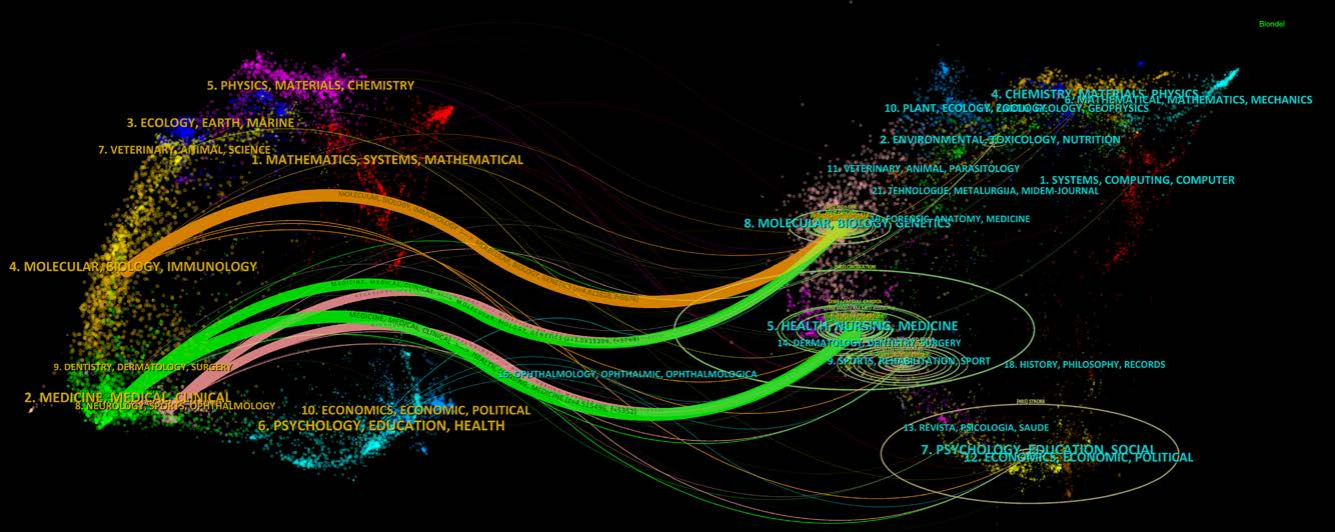
The biplot overlay of journals.

CiteSpace dual-map overlay function was used to construct the subject distribution of academic journals. The double map overlay of journals (Fig. 7) showed that research published in the Molecular/Biological/Immunological journals, the Medicine/Medical/Clinical journals, and the Neurology/Sports/Ophthalmology journals commonly quoted studies published in the Molecular/Biology/Genetics journals and Health/Nursing/Medicine journals. The citing journals appeared on the left side of the map, while the cited journals appeared on the right side.

### 3.6. Bibliometric analysis of co-occurring keywords

**Figure 8.**
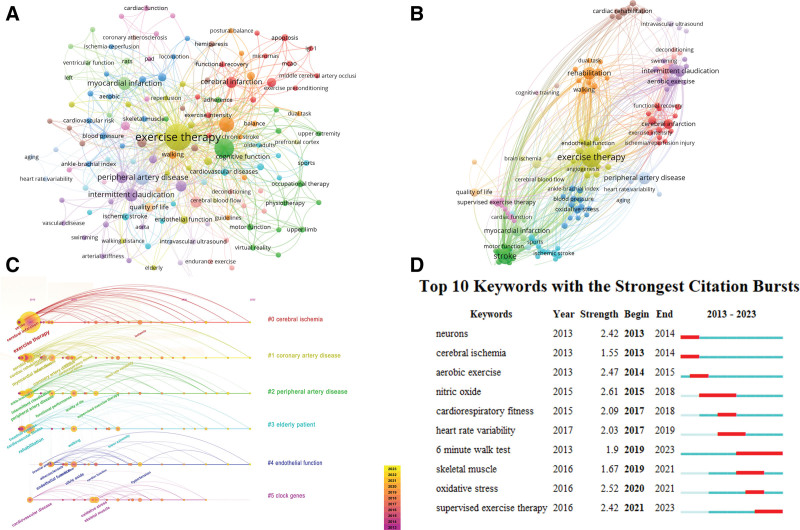
The mapping on keywords of exercise therapy for Panvascular disease. (A) Network diagram, (B) Keyword clustering graph, (C) Timeline map of the co-occurrence of keywords, (D) Top 10 keywords with the most robust citation bursts.

In this study, we used VOSviewer to analyze the co-occurrence of keywords in 294 publications across all documents. The network of keyword co-occurrences enabled us to identify research hotspots and trends in the field. 141 author keywords appeared more than 2 times (Fig. 8A and B and Table 6). The most frequently used keyword was “exercise therapy,” followed by “stroke,” “rehabilitation,” “peripheral artery disease,” and “intermittent claudication.” All keywords were classified into 6 clusters (#0 cerebral ischemia, #1 coronary artery disease, #2 peripheral artery disease, #3 elderly patient, #4 endothelial function, #5 clock genes) and identified by a timeline in CiteSpace, as shown in Figure 8C. The figure showed the top 10 keywords with the most vigorous citation bursts. Keywords burst detection revealed research hotspots over time and reflected the trend of hotspot evolution by identifying abrupt increases in frequency over a brief period (Fig. 8D). The blue line represented the time interval, while the red line indicated the period in which a keyword was found to have a burst. These keywords included 2 main categories: exercise therapy programs (e.g., aerobic exercise, supervised exercise therapy) and exercise therapy mechanisms (e.g., neurons, nitric oxide, heart rate variability, oxidative stress). In summary, recent research frontiers were supervised exercise therapy and oxidative stress.

**Table 6:** The top 10 keywords in exercise therapy for Panvascular disease.

Rank	Frequency	Keywords	Centrality	Keywords
1	155	Exercise therapy	0.77	Exercise therapy
2	40	Rehabilitation	0.18	Rehabilitation
3	35	Myocardial infarction	0.15	Coronary artery disease
4	32	Cerebral infarction	0.13	Myocardial infarction
5	28	Coronary artery disease	0.12	Cerebral infarction
6	27	Peripheral artery disease	0.09	Cardiovascular disease
7	25	Intermittent claudication	0.09	Functional performance
8	23	Cardiac rehabilitation	0.08	Peripheral artery disease
9	22	Aerobic exercise	0.08	Cardiac rehabilitation
10	18	Stroke	0.07	Nitric oxide

## 4. Discussion

### 4.1. General information

294 articles were retrieved from the Web of Science database from June 2013 to June 2023 on exercise therapy in Panvascular disease; these articles were published by 1858 authors, 642 institutions, and 42 countries. The number and trend of references published each year reflected a research field development, speed, and progress. The number of publications showed a trend of steady annual growth in the last decade. Mary M. McDermott has published the most articles. This author study focused on PAD. Another critical author, Gardner Andrew W, had the highest citation rate and showed an excellent academic influence. In addition, 4 of the top ten authors in terms of production were from China, corresponding to China being the most productive country in this field. Although the USA is narrowly behind in second place in the number of publications, it was far ahead in terms of the intensity of cooperation.

Meanwhile, 8 of the top ten co-cited authors were from the USA. Notably, China and Brazil performed well regarding the number of articles published. However, China and Brazil could be higher in terms of the intensity of cooperation, suggesting that the quality of research could be further improved. Most of the top ten producing institutions were in China, the USA, and the Netherlands. The University of São Paulo in Brazil has published the most research in this area, but there were relatively fewer citations, probably due to a lack of influential authors and international collaborative networks.

By analyzing the co-cited journals about exercise in Panvascular disease, we determined that researchers concentrated on the fields of the heart and cardiovascular system, peripheral vascular disease, and clinical neurology. The top 10 co-cited journal categories included sports science, internal medicine, rehabilitation, biology, and physiology. This finding suggested that applying exercise therapy in Panvascular disease often required multidisciplinary collaboration. In addition, from the keywords, clusters, and keywords with citation bursts, the primary research in this field focused on the application of different exercise therapies in Panvascular disease, the application of exercise therapy in various types of Panvascular disease, and the mechanisms of exercise therapy in Panvascular disease. Stroke was one of the most studied Panvascular diseases. The most recent frontier of exercise intervention was “supervised exercise therapy,” and the frontier of exercise mechanisms was “oxidative stress.”

### 4.2. Effect of exercise therapies on Panvascular disease

According to the keyword burst graph, there were 2 keywords related to the program of exercise therapy: aerobic exercise and supervised exercise therapy. In addition, the keyword analysis showed that keywords also included: treadmill exercise, robotic-assisted training, and underwater exercises. This suggested that applying different exercise therapies was still a hot research topic in this field. Aerobic exercise was widely used in Panvascular disease. Aerobic exercise could improve post-stroke patients’ quality of life and improve depression, endurance, and mobility.^[31]^ Aerobic cycling improved walking ability in stroke patients.^[32]^ Both aerobic interval and continuous training consistently improved peak oxygen uptake, peripheral endothelial function, cardiovascular risk factors, and quality of life in patients with CAD.^[33]^ High-intensity home-based walking exercise significantly improved the 6-minute walking distance in PAD patients.^[34]^ In addition, different exercise therapies had other effects on Panvascular disease. Aquatic walking reduced leg arterial stiffness and increased the 6-minute walking distance and peak oxygen uptake in patients with PAD.^[16]^ Supervised exercise therapy reduced mean systolic blood pressure, pulse pressure, and heart rate in patients with PAD, along with significant improvements in 6-minute and maximal treadmill walking distance.^[35]^ For patients with CAD, moderate-intensity water-based circuit training exercises could improve aerobic capacity, increase leg strength and reduce total body fat^[36]^ High-intensity interval training, moderate-to-vigorous intensity continuous training, and Nordic walking were all effective in improving CAD patients’ walking ability, quality of life, and depressive symptoms.^[37]^ Trunk stabilization training increased abdominal muscle thickness and improved balance and mobility in patients with hemiplegic stroke.^[38]^ Baduanjin exercise significantly improved limb motor function, balance, muscle strength, step length, walking speed, and cadence in patients with post-stroke cognitive impairment.^[39]^ Although different exercise therapies showed potent effects in Panvascular diseases. However, given the diversity of exercise therapies and the variety of Panvascular conditions, and Considering the adverse effects that could result from prolonged or short-term high-intensity,^[40,41]^ finding the best exercise therapy for patients with different types of Panvascular disease and clarifying the recommended dose and intensity of exercise for Panvascular disease required further in-depth research.

### 4.3. Potential mechanisms of exercise therapy in Panvascular disease

In addition, the keyword burst also showed foul keywords related to exercise mechanisms: neurons, nitric oxide, heart rate variability, and oxidative stress. This means that the search for the mechanisms of exercise therapy in Panvascular disease has been ongoing over the past decade. In 2013, research on the mechanisms of exercise therapy focused on “neurons.” Zheng et al showed that exercise increased the number of new neurons and neural progenitor cells and decreased apoptosis in the peri-infarct region following ischemic stroke.^[42]^ Nitric oxide had many biological properties that maintained vascular homeostasis, including anti-inflammatory, antioxidant, and anti-thrombotic and inhibited smooth muscle cell proliferation and migration.^[43]^ Exercise significantly increased serum nitric oxide levels; meanwhile, endothelial nitric oxide synthase was phosphorylated and activated during exercise.^[44,45]^ Aerobic training and high-intensity interval training reduced central arterial stiffness in humans and rats by increasing the bioavailability of aortic nitric oxide.^[46]^ A recent study showed that exercise pretreatment significantly induced downregulation of anti-oxidative stress markers, Heme oxygenase-1 and superoxide dismutase 1, expression in the peri-infarct cortex of mice, which may indicate that exercise could alleviate oxidative stress after stroke.^[47]^ Additionally, in a high-fat diet-induced apolipoprotein-deficient mice model of ASCVD, exercise-downregulated interleukin (IL)-6, IL-1β, intercellular adhesion molecule 1, vascular cell adhesion molecule 1, and monocyte chemoattractant protein 1 levels in aortic tissue. It reduced plaque area and lipid accumulation in plaques. Mechanistically, exercise promoted the level of methyl-CpG-binding protein 2 lysine lactylation in plaque endothelial cell nuclei, which regulated the activity of the epiregulin/mitogen-activated protein kinase signaling pathway, ultimately reducing the endothelial cell inflammatory response and attenuating plaque development in ASCVD.^[48]^ In a mouse model of ischemic stroke, exercise preconditioning reduced the proportion of M1 microglia and the expression of tumor necrosis factor-alpha and IL-6 in the peri-infarct cortex. It increased the proportion of M2 microglia and the expression of myeloperoxidase and IL-10. Expression of the pro-apoptotic proteins, autophagosomal autophagosome LC3II/LC3I ratio, and the autophagic substrate protein degradation marker in the peri-infarct cortex were also significantly reduced after exercise. In contrast, the expression of the anti-apoptotic protein and the lysosomal markers was enhanced. This evidence suggested that exercise ameliorated neuroinflammation, neuronal apoptosis, degeneration, and impaired autophagy and improved autophagic flux in ischemic stroke. Further experiments revealed that exercise pretreatment improved stroke prognosis by activating the AMP-activated protein kinase pathway, leading to transcription factor EB nuclear translocation.^[47]^ Exercise may have increased perfusion, arteriole density, and muscle regeneration in the ischemic hindlimb regeneration by inhibiting M1 macrophage polarization and inflammation in ischemic muscles and ultimately increased the maximum running distance and maximum running time of PAD model mice. After exercise, the macrophage marker, the number of arterioles, the number of regenerating myofibers, the percentage of anti-inflammatory monocytes, the expression of pro-arteriogenic genes, and myogenesis-related genes and quiescent satellite cells expressed the transcription factor were all increased in the ischemic muscle of mice, along with the percentage of pro-inflammatory monocytes and the levels of M1 macrophage markers nitric oxide synthase and IL-6 were decreased.^[49]^ These findings provided new insights into the role of exercise in Panvascular disease. Neurons, nitric oxide, and oxidative stress were the hotspots of research on the mechanisms of exercise intervention in Panvascular disease identified by Citespace analysis. The mechanisms of exercise intervention were very complicated, and future studies could delve into the interactions between different mechanisms.

## 5. Strengths and limitations

Our study conducted a comprehensive bibliometric analysis using Citespace and VOSviewer. This analytical approach facilitated the elucidation of critical domains of scholarly interest and the exploration of collaborative endeavors among nations, institutions, and authors. The dataset from the WOSCC database encompassed most articles germane to exercise therapy in Panvascular disease research. Through relatively objective and comprehensive data analysis, we gained insights into this field historical and contemporary landscape and ventured into prognosticating future frontiers of inquiry.

Nonetheless, it was vital to acknowledge the limitations present in this study. Firstly, our literature search was confined to the WOSCC database, which might have resulted in an incomplete literature search. Secondly, we exclusively focused on English language publications, as the primary indexing of the Web of Science database encompassed papers predominantly authored in English. This approach might need to pay more attention to relevant research published in other languages.

## 6. Conclusion

This study analyzed the countries, institutions, authors, journals, and keywords of research papers on exercise therapy interventions for Panvascular diseases published from June 2013 to June 2023. The results showed that research on exercise interventions for Panvascular illnesses had received wide attention and maintained continuous growth in the last decade. Research in this field is concentrated in China and the USA. The United States had a vast network of collaborations and many research institutions that dominated the area. Other countries should strengthen international cooperation and institutional affiliation to promote further development. Most publications in this field are related to the heart and cardiovascular system, peripheral vascular disease, clinical neurology, sports science, internal medicine, rehabilitation, biology, and physiology. They implied that greater interdisciplinary collaboration was essential for the development of the field. The keyword analysis suggested further exploring optimal exercise therapy programs and the mechanisms underlying exercise was necessary.

## Acknowledgments

Thanks to all study participants for their cooperation.

## Author contributions

**Conceptualization:** Lin Zhu, Kun Ren.

**Formal analysis:** Xi Xu, Yin Liang.

**Funding acquisition:** Fu Rong Shao, Lin Zhu.

**Methodology:** Xi Xu, Xiao Dan Xu.

**Software:** Xi Xu, Tao Xu.

**Supervision:** Kun Ren.

**Visualization:** Xi Xu, Fu Rong Shao.

**Writing – original draft:** Xi Xu, Xiao Dan Xu, Kun Ren.

**Writing – review & editing:** Lin Zhu, Kun Ren.
